# Ultrasonic force microscopy for nanomechanical characterization of early and late-stage amyloid-β peptide aggregation

**DOI:** 10.1038/srep04004

**Published:** 2014-02-06

**Authors:** Claire Tinker-Mill, Jennifer Mayes, David Allsop, Oleg V. Kolosov

**Affiliations:** 1 Physics Department, Lancaster University, Lancaster, LA1 4YB, UK; 2 Division of Biomedical and Life Sciences, Lancaster University, Lancaster, LA1 4YB, UK

## Abstract

The aggregation of amyloid-β peptides into protein fibres is one of the main neuropathological features of Alzheimer's disease (AD). While imaging of amyloid-β aggregate morphology *in vitro* is extremely important for understanding AD pathology and in the development of aggregation inhibitors, unfortunately, potentially highly toxic, early aggregates are difficult to observe by current electron microscopy and atomic force microscopy (AFM) methods, due to low contrast and variability of peptide attachment to the substrate. Here, we use a poly-L-Lysine (PLL) surface that captures all protein components from monomers to fully formed fibres, followed by nanomechanical mapping via ultrasonic force microscopy (UFM), which marries high spatial resolution and nanomechanical contrast with the non-destructive nature of tapping mode AFM. For the main putative AD pathogenic component, Aβ1-42, the PLL-UFM approach reveals the morphology of oligomers, protofibrils and mature fibres, and finds that a fraction of small oligomers is still present at later stages of fibril assembly.

There are several different neurodegenerative diseases, including prion disease, Alzheimer's disease, Parkinson's disease and Huntington's disease, in which a particular misfolded protein (or more than one protein) contributes over time to extensive neurodegeneration in specific regions of the brain. This leads to the corresponding clinical features of the disease in question. AD is the most common cause of dementia in the elderly; the number of cases in the US alone was 4.5 million in 2000, and this is set to triple to 13 million by 2050^1^, with 26.6 million cases being diagnosed worldwide in 2006^1^,^2^. AD is one of the most widely studied of the *amyloidoses*, a group of diseases in which the central pathogenic feature is the accumulation of insoluble *amyloid* fibrils, which are typically 7–10 nm in width, with a high β-pleated sheet content, and an ability to bind to the dyes Congo red or thioflavin T (ThT)^3^,^4^. As the main putative pathogenic component of AD, amyloid–β(1–42) (Aβ1-42) has attracted a great deal of interest. This protein follows a hierarchy of aggregation from a 4 kDa monomer, through small oligomers, to short flexible chains called protofibrils (PF), and finally to mature amyloid fibrils (MF) which appear to be derived from the twisting together of two or more PF to create a rope-like structure^5^,^6^,^7^,^8^. While it was initially thought that the senile plaques containing MF were the neurotoxic component in AD, research now suggests that that early stage aggregates are likely to be more damaging to nerve cells^6^,^8^,^9^,^10^.

Multiple methods are currently used to study the dynamics of Aβ aggregation, including ThT and Congo red binding^11^, size exclusion chromatography^8^, light scattering^7^,^8^, mass-spectroscopy^8^ and immunoassays^8^,^9^. Individually, these techniques provide specific information on one particular aspect of amyloid aggregation, such as β-sheet content, particle size or the availability of epitope binding sites. However, they do not allow for detailed study of overall morphology, or indeed, any alterations in morphology resulting from the structural transitions undergone during the pathway(s) leading from monomeric peptide, through oligomeric assemblies, to PF and MF structures. Such morphological knowledge, directly a result of the underlying structure, is vital for developing aggregation inhibitors as a potential treatment strategy for amyloid diseases and can only be obtained via nanoscale resolution microscopy methods, such as transmission electron microscopy (TEM)^5^,^12^ or atomic force microscopy (AFM).

Imaging of biological samples by TEM typically requires heavy metal staining. This can lead to cross linking between residues in the protein, distortion of the substructure, and can also mask nanostructural features. Imaging of unstained samples is limited to proteins of mass >100 kDa, which in itself proves to be problematic when trying to image very small structures. For this reason, Aβ oligomers containing <20 monomers would not be detected^13^. In contrast, imaging with AFM requires simple sample preparation and no staining, so samples are more reflective of their *in vivo*, native state. AFM imaging is, however, relatively slow, taking minutes rather than the seconds required for TEM. Additionally, AFM does not allow rapid alteration of the magnification and field of view that would facilitate finding the sparse features – a clear advantage of electron microscopy (EM). AFM also needs atomically flat, or at minimum, nanoscale-flat, rigid substrates, somewhat limiting its applicability to some studies, compared to TEM, where free standing membranes are widely used on “holey silicon nitride” or “holey carbon” TEM grids.

When using AFM one must also be careful with regard to the forces applied to the sample, as biological materials, such as proteins, are fragile and susceptible to tip-induced damage. As a result imaging of biological material with an AFM usually employs tapping mode (TM), which reduces contact with the sample and the sheer forces applied to it. Other limiting factors of AFM are the broadening of features due to the tip size, potential influences of the substrate surface on the sample and low contrast to internal features of larger structures, e.g. MF assemblies, as generally only the surface is measured.

AFM and TEM studies of aggregation have noted a number of different configurations of Aβ, including smooth or nodular fibres, amorphous globular aggregates, and PF structures ranging from 50–200 nm in length^8^,^14^,^15^,^16^. Early aggregation time points show that the protein has a globular structure while present as an oligomer. Once a critical mass has been reached, elongation occurs by the addition of monomeric or oligomeric units to the nascent end of each PF, with packing of the β-sheets stabilizing the structure^17^,^18^. Unfortunately, neither TEM nor AFM currently allow efficient imaging of the early, hypothetically toxic, stages of amyloid aggregation, due to the low contrast of nano-sized oligomers and PF. Additionally, the question of whether capture on current microscopy substrates represents all stages of amyloid aggregation still remains unclear.

Here, in order to address these issues, we present a method for the study of amyloids at various stages of their assembly that combines well established poly-L-lysine (PLL) coating on atomically flat mica surfaces^19^,^20^, with nanomechanical mapping using ultrasonic force microscopy (UFM)^21^,^22^,^23^. This unique combination offers new contrast, similar to teaming AFM with Raman or fluorescent imaging. While PLL allows the capture and retention of peptides, UFM provides natural contrast based on the non-specific, intrinsic, nanomechanical properties of protein assemblies, potentially providing information indicative of changes in underlying structure. When applying these techniques to protein aggregates there are a number of additional factors to consider. The PLL capture, while generally efficient, is not necessarily universal and may selectively capture some species skewing the observable distributions. One must be aware of the tip-surface interaction in UFM, which although reduced compared to C-AFM, applies higher forces on the sample, compared to TM-AFM or non-contact AFM, which remain the gold standard for least destructive AFM methods. Finally, although UFM may be less susceptible to adhesion artifacts than TM-AFM, its increased normal forces can modify the vertical scale of imaged features more than in TM-AFM, and in some cases can irreversible deform nanostructures if they have a low limit for plastic deformation.

The PLL-UFM combination was tested on the Aβ1-42 peptide, one of the most challenging members of the amyloid family to study. Freshly dissolved peptide was aggregated by incubation for various times in phosphate buffer (PB). This investigation first examines the use of PLL-mica as a substrate for protein capture, before a further more detailed discussion of the UFM technique. Subsequent sections focus on the external and internal morphology revealed by imaging Aβ1-42 with UFM. PLL-UFM allowed the reliable identification of the aggregated structures formed, from low molecular weight oligomers, to PF and MF, as well as revealing internal structural features of the latter. This new method can be used in the physiological environment and should be applicable to a broad range of studies of biopolymers and supramolecular structural morphologies.

## Results

### PLL-mica substrate for capture and nanomechanical imaging of peptides and amyloid aggregates

Nanomechanical mapping of low molecular weight peptides requires a sub-nm flat substrate. For AFM studies, samples are normally deposited directly onto mica substrates^18^,^24^. However, due to the similar isoelectric points of mica and Aβ1-42^11^,^25^ (both mica and Aβ1-42 are negatively charged at neutral pH) the attachment of this peptide to mica is likely to be problematic. A secondary problem is that the presence of salt within a buffer solution is essential for biologically relevant aggregation of Aβ1-42^17^,^26^,^27^. However, during the drying process of AFM samples, buffer salts can crystalize across the protein structures. This can interfere with the tip-surface interactions, while also obscuring topographical/nanomechanical details which are readily detectable once the crystals are removed. To overcome this problem, and to allow capture of diverse peptides, the mica was coated with dilute, low molecular weight, PLL solution (See Methods). This allows the peptide to interact electrostatically with the coated mica substrate, for an attachment that is strong enough to survive the subsequent washing procedure, with no defined background topography, as reported elsewhere^19^. It is important to note that when high concentrations of PLL are mixed with Aβ in solution, fibre morphology is disrupted^28^. However, our technique differs as the protein does not come into contact with the fibres in solution, because the PLL is baked on to the surface of the mica. In order to confirm that the PLL coated substrate did not cause any gross morphological changes to the protein following deposition, samples were monitored before and after exposure to the PLL surface using the ThT assay, and no gross alteration in β-sheet content was detected (data not shown).

**Figure 1:**
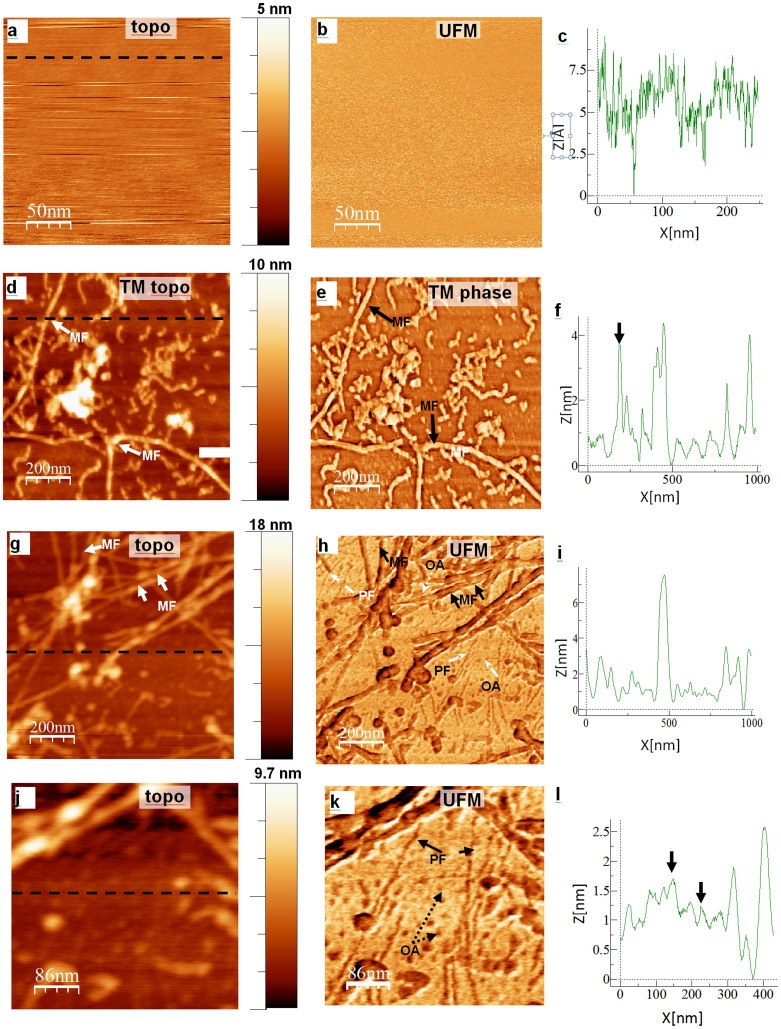
Comparison of AFM TM and UFM nanomechanical imaging of amyloid-β peptide aggregates. The control sample of PLL-coated mica was produced using the same protocol, but without adding rAβ1-42. (a) TM topography and (b) UFM stiffness mode shows a featureless flat surface; topography is close to atomically featureless with a 0.2 to 0.4 nm peak-to-peak noise floor. TM topography (d) and phase contrast (e) images of rAβ1-42 incubated for 72 h show short and long straight and curved mature amyloid fibres (MF) (solid arrows) of 3 to 6 nm high, as estimated by the z coordinate in (f). UFM topography (g) from the sample with the same incubation time shows similar height MFs (from profile (i), whereas a UFM stiffness map (h) clearly shows straight protofibrils (PF, dashed arrows) and round oligomer assemblies (OA, dashed arrows) that are not visible in the topography images; darker UFM contrast corresponds to lower stiffness. A high resolution image of a small section of the UFM topography/signal panel shows poor contrast in the topographical presentation (j) compared to the UFM (k) profile. (l) shows the approximate position and height of oligomers more clearly seen with UFM contrast. More than 80 oligomers are present within a 1 μm^2^ area, along with approximately 40 PF.

Using the PLL coating, we have observed greatly increased protein attachment compared to bare mica, allowing reliable subsequent imaging. Importantly, no background topography was seen when using PLL-coated mica (Fig. 1a–c), with local peak-to-peak roughness in the order of 0.2–0.4 nm, comparing well with the 1–2 nm hydrodynamic radius of the Aβ monomer^26^,^29^. The PLL surface was also very robust, with no damage due to scanning at moderate contact forces being observed. We have used stiff force modulation cantilevers to scratch through the PLL coating and provide a measure of the coating thickness of 3.5 ± 1.7 Å. More importantly for imaging with UFM, no nanomechanical contrast was seen in PLL-coated mica treated in an analogous manner to that of control PLL-mica, without any Aβ1-42 (Fig. 1b), suggesting its efficient use for further nanomechanical probing of peptide aggregate morphology.

The stiff, robust and close to atomically flat, PLL-coated, mica surface, (See Supplementary Fig. 1), allowed reliable capture of peptide aggregates, as observed in tapping mode AFM images (Fig. 1 d, e) as well as in UFM images (Fig. 1 g–k), to be discussed in detail below. All components from small oligomers, to PF and larger MF, remained attached to the mica following gentle washing, making this protocol highly efficient for imaging of Aβ1-42 over a wide range of aggregate sizes and time points. We further investigated the attachment of Aβ1-42 aggregates and confirmed that they were not simply artifacts of the buffer system by using an immunogold based imaging system, employing an antibody to Aβ (Supplementary Information, Fig. 2).

### UFM in biological applications

AFM studies of biological samples and biopolymers are typically performed using tapping mode (TM)^3^. TM is characterized by the absence of shear forces, as it only permits brief contact between the cantilever and the sample. It is therefore less destructive for soft biological samples than contact mode (CM)^30^. TM topography captures the shape of the sample, and TM phase contrast senses variations in local adhesion, but TM cannot directly measure nanomechanical stiffness in the same way as CM^3^ and TM has a lateral resolution that is generally inferior to that of CM. However, the drawback of CM is that it creates shear forces as the tip scans across in a raster pattern which can tear or otherwise damage the sample. In order to combine the benefits of both methods, we have used UFM, a technique that combines the sensitivity of acoustic microscopy^31^ with the mechanical properties and nanometre spatial resolution of CM.

**Figure 2:**
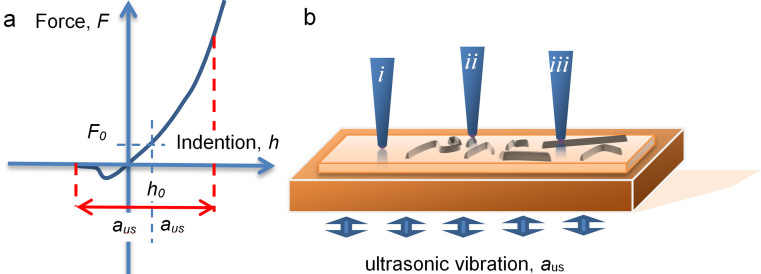
(a) Force-indentation curve for AFM operating in UFM mode^22^,^33^. The initial set force F_0_ results in the tip indenting the surface to the depth h_0_, usually on the order of sub-nm range. Ultrasonic vibration of amplitude a_us_ that is larger than indention h0 is applied to the sample (panel (b)) and results in the modulation of the instantaneous force, F. The instantaneous force while averaged over ultrasonic period T, differs from the initial force F_0_, due to the nonlinearity of force-vs-indentation curve F(**h**). This additional “ultrasonic” force creates static deflection of the cantilever, that depends on the slope of the F(**h**), and, therefore, elastic moduli of the sample under nanometre scale area of the tip^53^. (b) Schematic illustration of the tip Poly-L-Lysine (PLL) coated mica sample with the areas free of peptides (**i**), with the small oligomer aggregates (**ii**) and mature fibril (**iii**).

**Figure 3:**
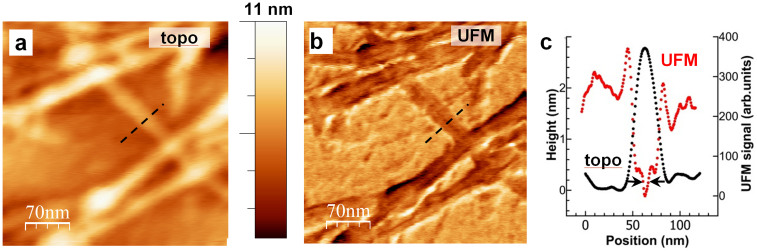
High magnification (a) topography and (b) UFM image of Aβ1-42 after 72 h *in vitro* incubation.(c) Corresponding one dimensional UFM stiffness profile (red dots) across the MF (dashed lines in (a) and (b) reveals internal structure invisible neither in the topography image nor in the topography profile (c), black dots) with the width of the softer region in the fibre center being approximately 5 nm (c), arrows in UFM profile).

UFM is well known for its superior performance in the mapping of nanoscale mechanical properties of stiff materials and shows potential for biomedical applicatons^22^,^23^. UFM uses a high frequency (few to tens MHz range) and small amplitude (sub-nm to few nm) ultrasonic vibration in such a way that a nanoscale tip is in contact with the sample for approximately half of the vibration period, reducing tip-surface shear forces^32^,^23^ (see Fig. 2). This makes possible elimination of damage to the peptide, common during contact imaging (Supplementary Methods and Results, Fig. 3 b,d), producing clear nanoscale morphological imaging of the peptide on the PLL substrate, as in Fig. 1 g,h and Fig. 3a. UFM therefore brings together the superior spatial resolution and nanomechanical sensitivity of CM with the non-destructive nature of TM.

### Topographical mapping of amyloid structures in UFM

In this study, Aβ1-42 aggregated for 72 h was imaged by both TM and UFM (Fig. 1 d–l). The TM topography and phase images (Fig. 1 d,e) showed elongated MF, with some smaller, curved structures. While UFM topography shows a similar level of detail to this (Fig. 1 g), the UFM stiffness image (Fig. 1h) is clearly able to reveal the presence of small oligomers and PF that are not seen by standard TM. It also offers insight into the elastic nanostructure of the fibre core and the fibre substructure, within the long, straight MF. MF are 4.15 ± 1.37 nm in height with an average width of 17.72 ± 8.85 nm, (n = 32), and are typically >1 μm in length. The width vs height difference in both TM and UFM images is linked with the convolution of the tip shape and the nature of the force interaction of the AFM tip with the top of the fibre, with both acting to increase the observed fibre width^3^,^33^. Typically, diameters of amyloid fibres are reported as a reflection of their heights. However, due to the stacking nature of the β-sheets it is probable that the fibril morphology represents a somewhat flattened cylinder shape, in which case height may not be an ideal reflection of diameter. Our width measurements therefore take this into consideration (see Methods).

### Mapping of nanomechanical internal structure of mature fibres (MF)

A closer look at the MF (Fig. 3) shows no particular details in a topography profile (both TM and UFM topography), while UFM shows an elastic property across the whole fibre width, and indicates that elasticity is not uniform across it. From the nanomechanical map of MF a discernible internal structure of the fibres is observed. Evidence of an internal morphology to the fibre structure has been presented previously using scanning tunnelling microscopy (STM) which indicated a domain texture to the fibres, while a hollow core within each fibre has been previously observed by cryo-EM^34^,^35^,^36^,^37^. More recent work has proposed the Aβ1-42 MF is formed from two peripheral regions surrounding a central core. When examined by cryo-EM this core contains a central region of lower density and two higher density packed cores^38^,^39^.

Nanomechanical analysis of the isolated MF profile (dashed line in Fig. 3b) suggests that these fibres are made up of 2 PF, each approximately 2–5 nm wide, with the central softer region of <1 nm full width at half maximum (Fig. 3c). It is possible that the central region detected is reflective of a hollow core left by the packing of the β-sheets and the H-bonding established between the backbones of the PF. The size of this central region indicates that the spatial resolution of UFM for mapping of nanomechanical properties is approximately 5 nm or better. While it is still debatable whether the Aβ1-42 MF is made of 2 or more protofibrils, we believe this softer region detected by UFM provides further evidence for a more complicated nanostructure to the protein, supporting the proposed MF conformation previously described by cryo-EM.

**Figure 4:**
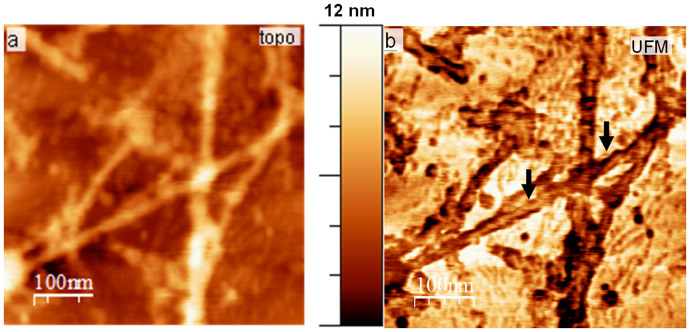
Detection of fibre twists with UFM compared to topography. More detail is seen on the UFM channel and changes in the nanomechanical properties of the fibre allow the twist to be readily visible, in comparison with topography. Periodocity is more detectable within the UFM channel output, and analysis suggests it to be approximately 25.7 ± 3.94 nm.

The nanomechanical imaging by UFM also revealed a periodic twist to the Aβ MF, a frequently observed criterion of Aβ fibre morphology. When present its periodicity is found to be approximately 25.7 ± 3.94 nm, (Fig. 4). The twisted morphology is visible in some fibres in the UFM channel as a variation in elastic properties of the fibre. While the topography frequently does not mirror this variation, it does indicate a slight decrease in height across a crossover point. While the width of the fibre does alter slightly across the cross sections, the distinct UFM profile does not, indicating this softer central region is detectable regardless of the twisting.

### Observation of *in-vitro* aggregation stages of amyloid structures

**Figure 5:**
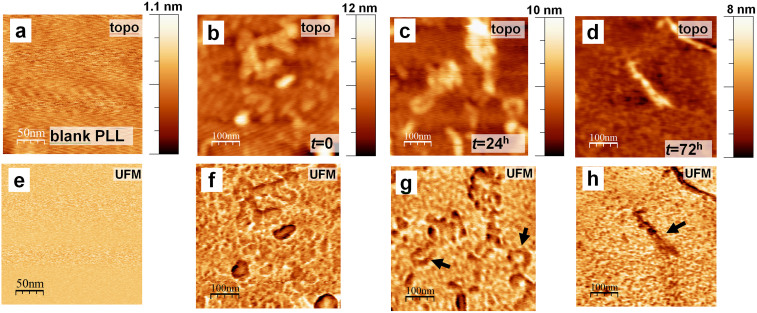
High resolution PLL-UFM mapping of progressive stages of *in vitro* aggregation of Aβ1–42. Reference topography (a) and nanomechanical map (e) of reference blank PLL-mica capture substrate shows no aggregates. Small oligomeric structures are clearly seen at t = 0 (b, f); elongated aggregates and short protofibrillar structures (arrows, (g) seen at t = 24 hours (c, g); developed fibres are clearly seen at t = 72 hours (arrow, (h), accompanied by a population of small and potentially toxic oligomers (d, h).

As we have observed, one of the key advantages of UFM is its ability to readily detect small aggregates of Aβ1-42. Immediately following solubilisation of the peptide, a layer of globulomers was detected on the mica surface (Fig. 5 b,f). On average these globulomers, as identified by UFM stiffness and measured at the same location in the UFM topography image, were 1.52 ± 0.61 nm (n = 892) in height, which is suggestive of values proposed by other teams for detection of the monomer and dimer-sized oligomers^16^,^26^,^29^,^40^. Larger features with a maximal height of ~6 nm are also noted and are proposed to be conglomerates of multiple monomers and dimers, in agreement with Lin and colleagues^40^. It was noted that oligomeric units, numbering >80 per 1 μm^2^ were still detectable at 72 h. Short flexible chains have also been detected throughout the course of aggregation and given their morphological and topographical features it seems likely that these represent PF. After 24 h incubation (Fig. 5 c,g), individual PF height was found to be 0.74 ± 0.53 nm with a width of 1.75 ± 0.9 nm (n = 30), while a similar population of PF was present at 72 h (Fig. 5d,h) with heights of 0.59 ± 0.31 nm and UFM measured width of 4.82 ± 2.15 nm (n = 30). Throughout the incubation, the lengths of PF ranged from approximately 50 to 200 nm.

## Discussion

PLL-UFM examination of Aβ1-42 samples has shown essential features of the nanomechanical substructure of the amyloid fibres. While most would agree that MF are made of 2 or more PF twisted together, the exact number of PF required is still considered controversial. UFM supports this, indicating that 2 or more PF of 2–5 nm diameter make the MF^18^,^24^,^41^,^42^. However, details on the internal structure of the fibre are sparse beyond identification of its presence^19^,^36^,^37^. The detection of the fibre core made by UFM (Fig. 3) clearly shows a varying stiffness region in the centre of the MF that is corroborated by STEM and cryo-EM data^11^,^34^,^35^,^36^,^37^,^38^,^39^,^43^.

The other major finding of this paper is that the PLL-UFM method allowed the observation of a population of oligomers and PF that are still present at later time points, when MF would be expected to be the predominant form present in the sample. Greater than 80 oligomeric moieties of varying sizes can be noted within a 1 μm^2^ area, alongside a smaller population of PF (n = 30). These oligomers and PF are frequently absent from TM topography and phase images, only becoming apparent in the UFM stiffness map and yet, appear to have the same morphology as structures seen earlier in the aggregation process.

This presence of oligomers and PF throughout the time course is very interesting; these morphologies are generally considered to appear early on in the aggregation process, then disappear as they are sequestered into MF over time. In contrast to the reversible nature of early oligomer and PF formation, MF are likely to be much more stable, with no appreciable levels of monomer dissociation^3^,^18^,^44^,^45^. However, it has been suggested by previous studies that the aggregation of Aβ into fibres is not the only pathway possible. Evidence suggests that there are possibly ‘off-pathway' populations of oligomers and PF which fail to aggregate further, or are possibly delayed in their aggregation. Given that early aggregates and oligomers are now considered to be the most neurotoxic form of Aβ, the presence of this off-pathway route to oligomer formation is particularly important because it since suggests that, *in vivo*, oligomers could be formed throughout the whole course of AD, exacerbating damage already present^10^,^14^,^46^,^47^.

The driving force behind the reported PLL-UFM approach was the need to monitor early stage Aβ aggregation and to map the morphology of hard to visualize, low molecular weight, peptide oligomers. We found that coating freshly cleaved mica with dilute PLL solution increases the electrostatic interaction between mica and the peptide and provides a close to atomically flat substrate, which can capture a wide range of peptide moieties potentially including early aggregates, oligomers, PF and MF fibres, and is suitable for subsequent nanomechanical identification via UFM. UFM mode allowed morphological imaging with good contrast and a nanomechanical resolution of 5 nm, comparable with that of the TEM. The measurements obtained using UFM are in agreement with previous work regarding monomer/oligomer size^16^,^26^,^29^,^40^ and also for that of the PF and MF^18^,^24^,^37^,^41^, while offering key advantages over the standard TM and CM AFM for imaging protein samples. This technique a) provides profound contrast to small aggregates, b) allows the study of internal features of PF and MF fibres with a resolution comparable to that of TEM, as well as c) preserving the non-destructive nature of TM AFM. Crucially for the particular peptide system under study, we found that a population of oligomers and PF persists at least up to 72 h after solubilisation and commencement of aggregation, which might shed new light on off-pathway routes, or indirect pathways of peptide aggregation that are otherwise unseen. Moreover, we believe that the method described has potential for much wider applications, for the study of a broad range of biopolymers, biomolecular assemblies and supramolecular structures.

## Methods

### Deseeding of amyloid β1-42

The peptide was deseeded to remove any preformed aggregates using a protocol adapted by personal communication from Manzoni and colleagues^48^. rAβ42, (rPeptide, Ultra-pure, HFIP, A-1163-2, >97% purity), was dissolved in 1 mL trifluoroacetic acid containing 45 μL of thioanisol, before being dried gently under a nitrogen stream. The deseeded protein was then treated using 2 ml of 1,1,1,3,3,3-hexafluoro-2-propanol (HFIP) and briefly vortexed and sonicated. The peptide was then split into working aliquots and dried by centrifugation under a vacuum, to give a final protein mass of 22.5 μg per sample. For aggregation experiments rAβ42 was dissolved at 25 μM in 10 mM Phosphate Buffer, pH 7.4. This buffer system was selected because it has been shown to be an ideal medium for supporting aggregation, without the presence of excessive salt concentrations^17^,^18^,^49^. It should be noted that physiologically-relevant chloride, magnesium and calcium ion concentrations do not necessarily impede aggregation, or alter the end point morphology, but can alter the kinetics noted. The concentration of Aβ was selected to ensure that it is above the critical micelle concentration of the peptide^24^, so that aggregation would proceed, replicating the pathology which can take decades to occur in brain tissue where the peptide has been reported to be in the nano-molar concentration range. The time course of rAβ1-42 aggregation was monitored using the ThT assay (Supplementary Information, Fig. 4). For this, triplicate samples (10 μl) were taken at the relevant time points and added to the wells of a black 96 well microtitre plate. The samples were then injected with 50 μl 15 μM thioflavin T in 50 mM glycine-NaOH buffer, pH 8.5, mixed and read 10 times over 2 mins at Ex λ = 450 nm and Em λ = 482 nm, using a Synergy 2 multilabel plate reader (Biotek). Samples were incubated over a time course up to 72 h at 37°C. Time points were taken as indicated and unless otherwise stated all experiments were conducted with 25 μM peptide. Unless otherwise stated all materials were from Sigma Aldrich.

### Preparation of the poly-L-Lysine protein capture and mapping substrate

Poly-L-Lysine (PLL) coated mica was prepared by incubating freshly cleaved mica (Agar scientific) with 0.01% 70–300 kDa MW Poly-L-Lysine solution (Sigma Aldrich), diluted 1:10 in dH_2_O for 5 min before baking for 1 h at 60°C. Sample collection for imaging was initially performed by diluting a sample of the desired time point 1:10 in dH_2_O, aliquoting 2 μl of this onto prepared PLL-coated mica and allowing it to air dry. Once dry, samples were washed twice with 200 μl dH_2_O. The thickness of the PLL coating on the mica was determined by utilising the AFM (Supplementary Fig. 1) by applying increased force in the range 100–200 nN using higher stiffness cantilever (3 Nm^−1^, Budget Sensors) and mechanically removing the layer of PLL to reveal the mica beneath. The corresponding height of the PLL coat was then measured by the difference between the pit and the mica surrounding it. A control sample of PLL-coated mica was produced using the same protocol, but without adding rAβ1-42.

### Scanning probe techniques: tapping mode (TM) and ultrasonic force microscopy (UFM)

Samples were imaged using a Multimode NanoScope IIIa scanning probe microscope (Bruker AXS). Scan speeds were between 0.5–1.5 μm/s. UFM operation^22^,^33^,^50^ was implemented by introducing a piezoceramic actuator (4 MHz thickness mode resonance, PI) beneath the sample excited by function generator (LXI Keithley) at 4.2 MHz ultrasonic frequency modulated at 2.3 kHz as described elsewhere^33^. The UFM signal at modulation frequency (Supplementary Fig. 2) was collected by a lock-in amplifier (SR830, Stanford Research Systems). UFM imaging was performed using 0.2 Nm^−1^ tips, TM imaging using 40 Nm^−1^, 300 kHz resonance (Tap300-G, Budget Sensors) performed using contact mode AFM cantilevers (Budget Sensors, Contact-G, *k* = 0.2 Nm^−1^, both with an average tip radius of *ca.* 10 nm.

As AFM topography can be affected by convolution caused by the tip size during scanning, which is more significant when imaging small, soft samples. An estimation of the real size of the structure if treated as spherical/cylindrical can be made using the following equation: 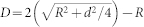


Where *D* is the real diameter of the object, *R* is the tip radius and *d* is the diameter measured at half the molecules height^51^.

All image processing was performed with WsXM^52^.

## Author Contributions

O.V.K. suggested the general idea for the method, J.M. performed pilot experiments of Aβ-imaging, C.T.M., D.A. and O.V.K. co-developed the capture methodology, C.T.M. and O.V.K. refined the imaging methodology, D.A. and C.T.M. developed model of Aβ system and interpreted the image data. All authors prepared the manuscript.

## Supplementary Material

Supplementary Information Supplementary informaion
