# Supplementary material for: Ultrasonic force microscopy for nanomechanical characterization of early and late-stage amyloid-β peptide aggregation
Source: Sci Rep. 2014 Feb 6;4:4004. doi: 10.1038/srep04004 (PMC3915309; doi:10.1038/srep04004)
Supplement: Supplementary Information — Supplementary informaion [file srep04004-s1.pdf]

Ultrasonic force microscopy for nanomechanical characterization of early and late-stage amyloid-β peptide aggregation

Claire Tinker-Mill[1], Jennifer Mayes[2], David Allsop[2], and Oleg V. Kolosov[1]

Supplementary Information.

**Supplementary Figure 1**	Determination of the thickness of the PLL layer.
**Supplementary Figure 2**	Immunogold staining of Aβ1-42
**Supplementary Figure 3**	Ultrasonic vibration induced elimination of friction and surface damage in nanoscale imaging of peptides in AFM.
**Supplementary Figure 4**	Thioflavin T data
**Supplementary Discussion 1**	Discussion on internal structure of Ab1-42 protofibrils (PF) vs mature fibres (MF); presence of off-pathway PF aggregates
**Supplementary Methods 1.**	Immunogold staining using a sandwich ELISA

Supplementary Figure 1. Determination of thickness of PLL layer.

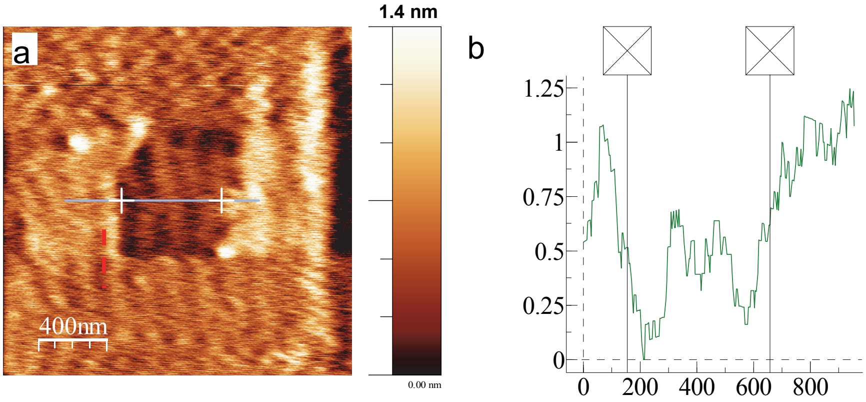

Supplementary Figure 1. Higher stiffness force modulation cantilevers (k = 3 Nm^{-1}) were used at elevated force of 100 nN to scratch the surface of PLL in contact mode AFM with no ultrasound over the 500x500 nm^2 square area (**a**). **b** The profile across this area indicated the typical thickness of PLL layer of 0.36 ± 0.15 nm providing robust and close to atomically flat substrate for nanomechanical UFM imaging.

Supplementary Figure 2. Immunogold staining of Aβ1-42

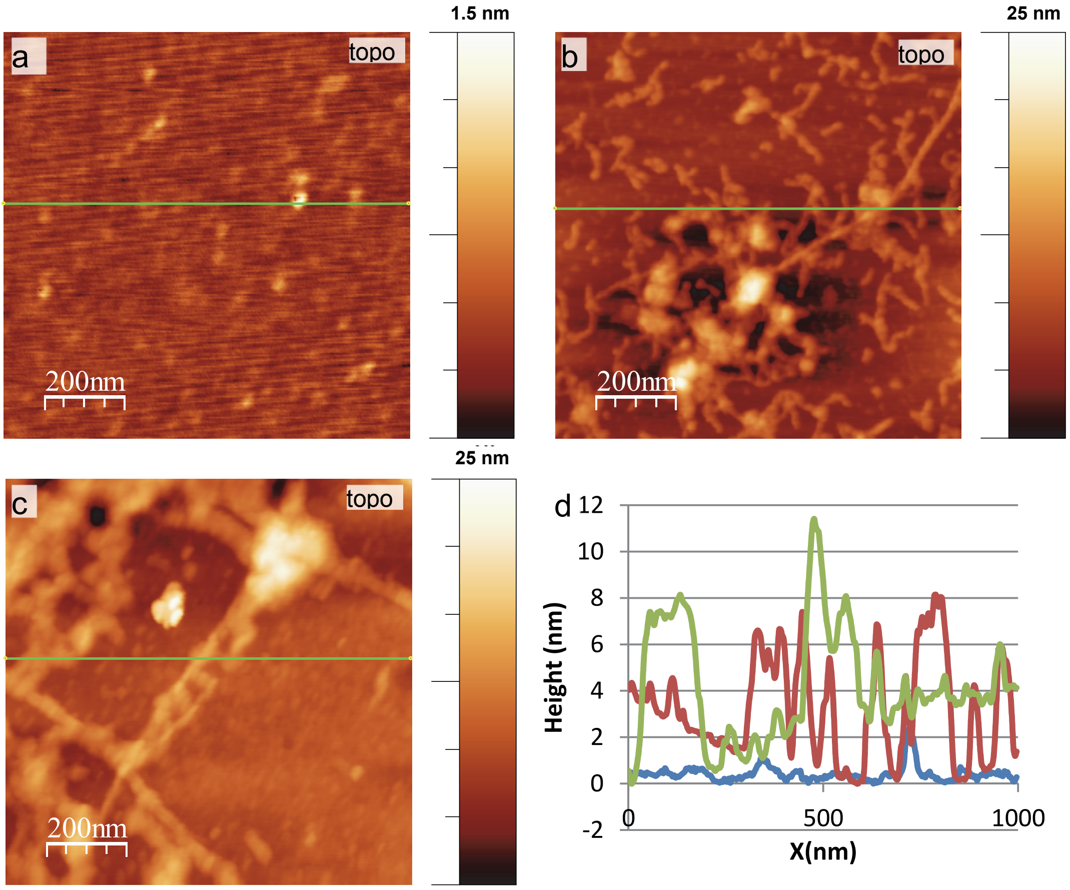

Figure 2. In order to ensure that the protein attached to the PLL-mica is indeed Aβ1-42 immunogold staining of the sample has been carried out. By adapting a sandwich ELISA containing AuIgG antibodies detection of small and large aggregates of Aβ is possible. Panel (a) PLL mica which has not been exposed to protein but has been treated with the full ELISA protocol, and is therefore a representative control.Pannel (b) shows the sample before staining, and (c) shows following. An appearance of a coating across the sample would indicate the presence of many small aggregates of Aβ. This is indicated by the increased width observed between fibres and aggregates in panels (b) and (c). An increase in height is also noted in the roughness profiles in panel (d), showing control (blue), before staining (red) and after staining(green).

Supplementary Figure 3. Ultrasonic vibration induced elimination of friction and surface damage in nanoscale imaging of peptides in AFM.

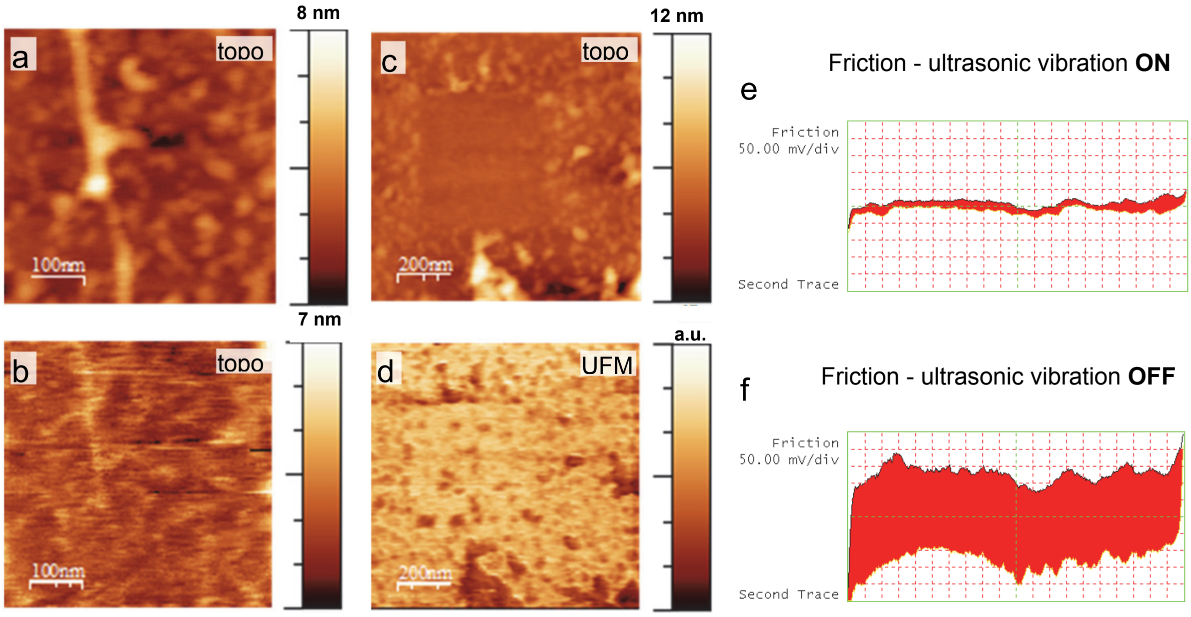

Figure 3. (a) Topographical image of amyloid fibrils and oligomer aggregates in the presence of ultrasonic vibration (UFM mode) and (b) a subsequent topographical image of the same area without ultrasound (standard contact AFM mode). Image obtained without ultrasonic vibration is much hazier with multiple streaks. (c) Topography and (d) UFM nanomechanical scans of the wider 1 μm area clearly showing that the 0.5 μm square area previously scanned in standard AFM contact mode (panel (b)) is grossly damaged with flattened topography in the square and most of the amyloid aggregates removed as seen in the UFM scan (d). While UFM preserves average tip-surface forces and therefore is capable of effective mapping of nanomechanical properties of peptides, as shown in the paper, its unique gentle non-destructive behaviour in imaging of biomaterials is linked with the drastically reduced friction during the ultrasonic vibration[1] (raw friction data, panel (e) compared with large friction (panel (f) in the standard AFM contact mode.

Supplementary Figure 4. Thioflavin T aggregation data

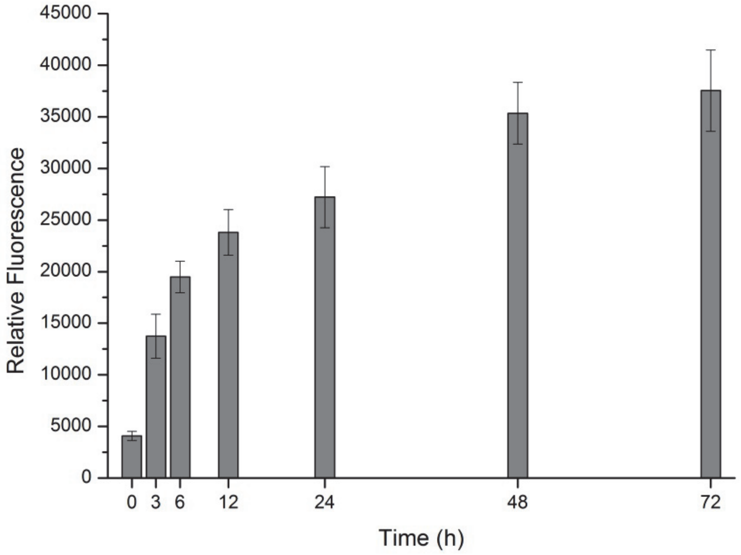

Supplementary Figure 4. Aggregation of Aβ1-42 was monitored using Thioflavin T assay in order to determine the presence of β-sheets within the sample. Samples were monitored at the above timepoints, (hours). Data is representative of at least *n=3* experiments.

Supplementary Discussion 1. Discussion on internal structure of Aβ1-42 protofibrils (PF) vs mature fibres (MF) and presence of off-pathway PF aggregates.

Each PF is made of Aβ1-42 monomers, each of which forms a β-sheet using residues 18-23 and 28-33 with residues 24-27 creating a connecting turn[2]. Monomer-oligomer attachment generates a conformational change within the β-sheets similar to that of closing a venetian blind[3]. These orientate perpendicular to the fibril axis and elongate to form β-sheeted strands, 2 of which pack face to face to from a double layered β-sheeted PF[4-6]. The structure is stabilised by hydrogen bonds in the β-sheet and also salt bridges between critical residues[6,7]. It is possible that the inner region of lower elasticity detected is reflective of the bonding between the individual β-sheet backbones. This leaves a hollow core as a matter of course and a similar situation is noted within the larger MF. The fact that this internal stiffness is absent from narrower PF suggests that a critical size is needed for individual β-sheets to interact to create a PF. It was observed that MF at earlier time points, notably fibres formed after 48 h incubation, were more susceptible to potential manipulation by the cantilever tip, than fibres seen at 72 h. Fibres before 72 h also appear to have a less rigid morphology than those sampled at later time points. This could indicate an increased compaction of the β-sheets at 72 h compared to earlier times, possibly

stabilising the MF structure and further increasing the protein's insolubility, an attribute which is currently under investigation.

Off-pathway PF aggregates have already been noted in other studies[8,9] but conflicting results exist as to whether they are a permanent or transient conformation for the peptide[10,11]. Theoretically, off-pathway moieties should be able to remodel their conformation into that which promotes aggregation. If this were the case, they could sequester neurotoxic oligomers which are slowly released over a much longer timespan than on pathway aggregates which are rapidly converted to fibrils[10]. However one should also consider the experimental data which suggests dissociation of oligomers/monomers from MF does not occur at appreciable levels, and that binding is typically irreversible[12-15]. Alternatively, the extended presence of PF which have not become incorporated into MF could simply be representative of a linear colloidal dispersal, a theory which has already been proposed[16]. The suspension of Aβ1-42 is highly reflective of a colloidal dispersion, in which the Brownian motion of the monomer/oligomer is essential for favourable collisions and subsequent aggregation. Over the time course, favourable collisions between the remaining oligomers leading to PF elongation, or the collision of 2 PFs and their subsequent twisting into a MF, will become less frequent. It is therefore perhaps not unsurprising that some PF remains visible after an extended period of time.

Supplementary Methods 1. Immunogold staining using a sandwich ELISA

Samples of peptide on PLL-mica were taken as previously mentioned, (25 μM Aβ42 and in 10 mM PBS, pH 7.4, incubated for up to 72 h), and placed in 12-well microtitre plates, (Sigma-Aldrich, Dorset, UK) and the samples were blocked with PBS plus 0.05% Tween-20 (PBST), for 15 mins. Samples were then coated with 6E10, diluted 1:1000 in 10 mM PBS, pH 7.4, for 1 h at 25ºC. The plates were incubated for 1 h at 37ºC and then washed with PBST. A 1:1000 dilution of the gold nanoparticle tagged IgG, (Aurion, Netherlands) was then added to each well, left for 1 h at 25ºC, and the plates were washed with PBS, and further washed with dH_2O. Gold nanoparticle size was 6nm. Samples were imaged once dry as described in the manuscript methods using Tapping Mode AFM.

References

1 Dinelli, F., Biswas, S. K., Briggs, G. A. D. & Kolosov, O. V. Ultrasound induced lubricity in microscopic contact. *Applied Physics Letters* **71**, 1177-1179 (1997).

2 Hard, T. Protein engineering to stabilize soluble amyloid beta-protein aggregates for structural and functional studies. *Febs Journal* **278**, 3884-3892 (2011).

3 Hoyer, W. & Hard, T. Interaction of Alzheimer's A beta peptide with an engineered binding protein - Thermodynamics and kinetics of coupled folding-binding. *J. Mol. Biol.* **378**, 398-411 (2008).

4 Di Carlo, M. Beta amyloid peptide: from different aggregation forms to the activation of different biochemical pathways. *European Biophysics Journal with Biophysics Letters* **39**, 877-888 (2010).

5 Karsai, A. *et al.* Mechanical manipulation of Alzheimer's amyloid beta 1-42 fibrils. *J. Struct. Biol.* **155**, 316-326 (2006).

6 Petkova, A. T. *et al.* A structural model for Alzheimer's beta-amyloid fibrils based on experimental constraints from solid state NMR. *Proc. Natl. Acad. Sci. U. S. A.* **99**, 16742-16747 (2002).

7 Guo, M., Gorman, P. M., Rico, M., Chakrabartty, A. & Laurents, D. V. Charge substitution shows that repulsive electrostatic interactions impede the oligomerization of Alzheimer amyloid peptides. *FEBS Lett.* **579**, 3574-3578 (2005).

8 Gellermann, G. P. *et al.* A beta-globulomers are formed independently of the fibril pathway. *Neurobiol. Dis.* **30**, 212-220 (2008).

9 Kumar, S. & Udgaonkar, J. B. Mechanisms of amyloid fibril formation by proteins. *Curr. Sci.* **98**, 639-656 (2010).

10 Powers, E. T. & Powers, D. L. Mechanisms of protein fibril formation: Nucleated polymerization with competing off-pathway aggregation. *Biophys. J.* **94**, 379-391 (2008).

11 Wetzel, R. Kinetics and thermodynamics of amyloid fibril assembly. *Accounts of Chemical Research* **39**, 671-679 (2006).

12 Gosal, W. S., Myers, S. L., Radford, S. E. & Thomson, N. H. Amyloid under the atomic force microscope. *Protein and Peptide Letters* **13**, 261-270 (2006).

13 Blackley, H. K. L. *et al.* High resolution investigations of beta-amyloid fibrillization by atomic force microscopy. *FASEB J.* **13**, A1574 (1999).

14 Lomakin, A., Chung, D. S., Benedek, G. B., Kirschner, D. A. & Teplow, D. B. On the nucleation and growth of amyloid beta-protein fibrils: Detection of nuclei and quantitation of rate constants. *Proc. Natl. Acad. Sci. U. S. A.* **93**, 1125-1129 (1996).

15 Lomakin, A., Teplow, D. B., Kirschner, D. A. & Benedek, G. B. Kinetic theory of fibrillogenesis of amyloid beta-protein. *Proc. Natl. Acad. Sci. U. S. A.* **94**, 7942-7947 (1997).

16 Carrotta, R., Manno, M., Bulone, D., Martorana, V. & San Biagio, P. L. Protofibril formation of amyloid beta-protein at low pH via a non-cooperative elongation mechanism. *J. Biol. Chem.* **280**, 30001-30008 (2005).
